# Progress in Diseases of the Blood

**Published:** 1903-07-18

**Authors:** 


### Progress in Diseases of the Blood.

**Human and Bovine Tuberculosis.**—The controversy as to their identity is rapidly becoming a repetition of that on the identity of human and bovine variola and promises to be as interminable. Baumgarten, who some years ago injected six persons dying of cancer with bovine tuberculosis and found them immune, comes forward with fresh evidence in support of Koch's theory of diversity.[1] Bovine tubercle, he says, chiefly affects serous membranes instead of the parenchyma of organs, it forms strings of large pearl-like nodules, and has a greater tendency to calcification. Theobald Smith has indeed shown that in cultures on blood serum bovine bacilli differ from human, and have a greater pathogenic power, though their morphological differences diminish when grown on other substances. Baumgarten, like Koch, claims that human tuberculosis cannot be transmitted to calves, but on this point experiments on a huge scale are being made in Germany. They both insist that neither by feeding nor by injection into the veins or peritoneum can reliable results be obtained, but only by subcutaneous injections. Even these, if very large, may cause intravenous emboli and unreliable results. As to the transmission of the bovine disease to human beings, Koch lays stress on the rarity of primary intestinal lesions in man, though he confesses they seem more common in England; and on the absence of endemics where many persons have fed on the same infected food. Ravenel[2] points out in reply that infection from food is often through the tonsils, that bacilli can pass through the intestine without causing any lesion and are carried to the mesenteric glands and thence into the lungs, where possibly the first lesion may occur, that no weight can be placed on the greater enlargement of the bronchial glands as showing the primary infection, and he adds that in England a quarter of all cases of tuberculosis seem to be due to infection through the intestinal tract. Ravenel's own experiments by the injection of cattle with human bacilli are most convincing, but are ruled out of court because they were intravenous or intraperitoneal. He especially sought for bacilli in cases due to food infection, since he thought that, if derived originally from the cow, they would be more pathogenic for that animal. This seemed to be the fact, and he showed that bacilli of a purely human ancestry increased in virulence when passed through a series of cattle. Besides this he refers to several well authenticated cases of the bovine disease which were inoculated into men, and concludes that on the whole human and bovine bacilli are of the same species, though the latter is more virulent. Fibiger and Hensen[3] have also injected successfully eight cattle with human bacilli, and five of these seem to have been subcutaneous injections. Arloing[4] also claims that he has infected 23 animals, four of them being oxen, but Koch's followers reject his results on the ground of the injections being intravenous, while Schottelius with minute precautions infected three cattle by mixing human sputa with their food.[5] Finally, in Great Britain an apparently decisive result has been obtained, which Koch and the dualists can hardly take exception to. Hamilton and Young,[6] in a series of experiments, infected not only four calves by feeding them with tuberculous sputum, but also five by subcutaneous injections. They found, too, that in the fed calves the bacilli had caused no lesion in the intestinal mucous membrane, though the mesenteric glands were enlarged; next, that human bacilli gained in virulence if passed through successive animals; and, finally, the possibility of thus passing on the disease to other animals showed that the lesions in those first injected were not due to dead bacilli as the dualists have suggested. These experiments were all carried out with the most minute precautions, the calves and the milk on which they were fed being previously tested, and their dwelling place newly built and spotlessly clean. As to the opposite side of the question, the inoculation of human beings by accident, such as of butchers from their work, Koch thinks all the recent evidence inconclusive (*vide* Berlin address), but Spronk and Koefnagel[7] have brought forward a case of a man who inoculated his finger in cutting up a tuberculous beast, and suffered from an induration and ulcer, with finally a large gland at the elbow. Portions of the tissues and of the gland were injected into guinea pigs, one of which then died of tuberculosis. Others were infected by inoculation from it, and finally a calf which had been previously tested. Thus bovine tuberculosis was conveyed to man and back again to the cow. Ravenel also reports a similar case.

The final proof of the conveyance of bovine disease to men by the food products of tuberculous cattle which is the practical side of the question is in strict logic still open to discussion. Nathan Raw[8] brings forward a theory which would explain some of these results. He acknowledges the distinctive peculiarities of cultures of bovine bacilli from those commonly obtained from man, but he thinks that tabes mesenterica, lupus, and probably tuberculous joint affections, and strumous glands are due to bovine tuberculosis, while phthisis generally arises from the true human form. He finds that cultivations from joints and from tabes show the characters of the bovine bacilli. Now while this theory would explain some of the peculiarities of local lesions in the skin, joints, and peritoneum, it would be necessary to show how the presence of such local infections or their toxins so frequently leads to pulmonary trouble. Moreover, bovine tuberculosis seems to have a greater [pathogenic power, and yet many of these local lesions are remarkable for their chronic course, and, indeed, if, as the above experiments seem to show, the two bacilli can be both grown in men or animals, it is hard to imagine that they are specifically distinct.

**Gelatine in Hæmoptysis.**—Grunow[9] employed these injections for arresting bleeding in phthisis, gastric ulcer, typhoid, and other affections, and reported favourably of it, though he noticed the pyrexia produced by it, and the pain at the site of the injection, and sometimes an urticarial rash. He employed a mixture made of 2 per cent. gelatine in saline solution and injected 200 cc. in one or two places, and found it necessary to continue the injections for some time after the bleeding had ceased. Two serious results have been noticed; cases of tetanus and cases of thrombosis have arisen in connection with this procedure. Levy and Bruns[10] examined six samples of commercial gelatine and found tetanus bacilli in four of them. From their experiments it would seem that the bacilli could not be got rid of by filtering through a Chamberland filter, though this seems hardly credible; but it is possible to get rid of them by prolonged boiling, which does not seem to destroy the hæmostatic power of the gelatine. As to thrombosis A. E. Wright points out that care must be taken not to carry the introduction of gelatine too far. The blood ought to be tested from time to time as to its rate of coagulation. The other objections are met by using rectal instead of hypodermic injections. H. M. Tickell[11] reports 20 cases in which this method was used with success. Fifty grammes of gelatine are dissolved in $1\frac{1}{4}$ litre of water and boiled gently for an hour when the volume is reduced to 1 litre. A quarter of a litre (9 ounces) is slowly introduced into the rectum three times a day by a tube from a vessel not more than three feet above the patient. This is done until the sputum is quite free from blood. There is no pyrexia or pain, and the treatment can be carried out easily in general practice.

**The Biological Test for Blood.**—A remarkable development of what is commonly called the Widal reaction is a test by which human blood can be detected even in dried stains or splashes and distinguished from that of animals. Spectroscopic and other tests in forensic medicine are far short of what is needed, and the new test promises to be of value in many cases. When an animal has been several times injected with serum drawn from another species, its own serum acquires the power of reacting to the blood of that species alone by giving "a præcipitum." So delicate is this that a blood can be detected when mixed with that of other animals, even when highly diluted; human milk can be distinguished from that of animals, and even bloods which have been kept for months in a dry or moist and septic state give the reaction. Thus the bodies causing the precipitation are remarkably stable. Noble[12] found that human blood-stains give the reaction up to two months, and that heating up to 100°C. did not destroy it but that if heated to 125° C. it failed. G. H. F. Nuttall and Dinkelspiel[13] extend and confirm the results of other workers, but notice that serum which reacts to a given species also affords a slighter reaction with closely allied ones, and the certainty of the results depends on the definite strength of the "anti-serum" and on a fixed time limit being taken. Thus while human blood can be separated from that of any animal, a slight reaction for this "anti-serum" can be also obtained with the blood of monkeys. Though the "anti-horse" serum reacted only with the blood of the horse and ass out of 500 animals, and the "anti-lobster" serum only with that of the lobster the crayfish and five species of crabs out of 500 tests, yet with stronger "anti-sera" or a longer time some clouding can be obtained in all mammalian blood by the serum peculiar to any mammal, but none with avian serum. Thus they find that a measure of the blood relation of different species can be formed. No difficulty from the legal point of view arises from the fact that human blood and serum from blisters and pleuritic fluid all give some reaction with the "anti-human" serum, for the reactions differ in degree, and under the quantitative methods introduced they can be easily measured. Indeed "anti-sera" can be formed from various fluids of the human body which react to one of those fluids in preference to others. Thus Martens and others, noticing that "anti-sera" formed by injecting albuminous urine reacted with blood, were induced to argue that the albumen must be derived from the blood. This of course has long been a debated point in renal pathology, but their conclusions do not seem quite unassailable. An immense number of tests have been made of such things as blood-stains on fabrics, and on weapons collected in Scotland Yard, also of blood mixed with grease, earth, mortar, and disinfectants. A few of the more powerful disinfectants, such as $HgCl_2$ and quicklime seem to prevent the action of the test, but it has been found possible to remove the lime in many cases, and generally to detect blood in the other conditions mentioned. Graham Smith and Sanger[14] have worked out the quantitative effects of age, heat, and contact with various substances upon human blood, and say that in few conditions met with in forensic practice would there be any difficulty in detecting it. It was found that human blood could be distinguished from animal in a stain 30 years old.

[1] Jour. of Tuberculosis, January. [2] Univ. Pennsylvania Bull. May 1902. [3] Berlin. Klin. Woch., No. 38, 1902. [4] Bull. de l'Acad. de Méd., Dec. 24. [5] Munch. Med. Woch., No. 39, 1902. [6] The Hospital, May. [7] Birmingham Med. Rev., January. [8] Brit. Med. Jour., Jan. 31. [9] Berlin. Klin. Woch., Aug. 12. [10] Deutsche Med. Woch., Feb. 20. [11] Lancet, Feb. 28. [12] Gould's Year-book, p. 591. [13] Jour. of Hygiene, Vol. 1 and Vol. 3. [14] Jour. of Hygiene, Vol. 3, p. 258.

